# Immune-Mediated Necrotizing Myopathy With Anti-3-Hydroxy-3-Methylglutaryl-CoA Reductase (HMGCR) Antibodies Following Viral Infection and Without Association With Statin Use: A Case Report

**DOI:** 10.7759/cureus.70281

**Published:** 2024-09-26

**Authors:** Pedro Ferreira, Francisca Dâmaso, Marta Anastácio, Fausto Pinto, Ana Lynce

**Affiliations:** 1 Internal Medicine, Hospital de São Francisco Xavier, Lisbon, PRT; 2 Physiopathology, Lisbon School of Medicine, Lisbon, PRT

**Keywords:** anti-hmgcr antibodies, immune-mediated necrotizing myopathy, internal medicine, reumatology, viral infection

## Abstract

Immune-mediated necrotizing myopathy (IMNM) is a rare form of inflammatory myopathy characterized by severe muscle weakness, elevated serum creatine kinase (CK) levels, and myofiber necrosis with minimal inflammatory infiltrates. IMNM is frequently associated with autoantibodies, particularly anti-3-hydroxy-3-methylglutaryl-CoA reductase (HMGCR), and is often linked to statin use. However, it can also develop in statin-naïve patients, especially following viral infections. We present the case of a 47-year-old woman who developed anti-HMGCR-positive IMNM without prior statin exposure, following a viral respiratory infection and subsequent dengue fever. She initially presented with proximal muscle weakness and elevated CK levels, which worsened after contracting dengue. Diagnostic testing confirmed the presence of anti-HMGCR antibodies, and a muscle biopsy revealed necrotizing myopathy. Treatment with methylprednisolone, intravenous immunoglobulin, and rituximab resulted in significant clinical improvement. This case underscores the need to consider IMNM in patients with unexplained muscle weakness and elevated CK levels, even in the absence of statin use. Viral infections may trigger IMNM, highlighting the importance of early recognition and aggressive immunosuppressive therapy to prevent severe complications. Further research is required to better understand the pathophysiology of IMNM and optimize treatment approaches.

## Introduction

Immune-mediated necrotizing myopathy (IMNM) is a distinct subgroup of inflammatory myopathies characterized by severe muscle weakness, elevated serum creatine kinase (CK) levels, and myofiber necrosis with minimal inflammatory infiltrates on muscle biopsy [1-3]. According to the criteria of the European Neuromuscular Centre, IMNM can be divided into three subtypes: anti-3-hydroxy-3-methylglutaryl-CoA reductase (HMGCR) IMNM, anti-signal recognition particle (SRP) IMNM, and antibody-negative IMNM [1,4]. IMNM may also be associated with malignancy or other connective tissue disorders [4,5].

For patients positive for autoantibodies (anti-SRP or anti-HMGCR), elevated CK levels and proximal muscle weakness are sufficient for diagnosis. In autoantibody-negative patients, a muscle biopsy is required to confirm the characteristic features of necrotizing myopathy [1,6].

Although rare, there is a recognized association between statin use and immune-mediated necrosis, distinct from other forms of drug-induced toxic myopathies. Unlike toxic myopathies, statin-induced IMNM does not improve with statin discontinuation, requires aggressive immunosuppressive treatment, and has been linked to anti-HMGCR antibodies since 2010 [1-7]. While the pathophysiology remains unclear, statin exposure, genetic predisposition, and viral infections are considered potential risk factors [1,3,7-10].

We present the case of a 47-year-old woman diagnosed with anti-HMGCR IMNM without prior statin exposure, following a viral infection.

## Case presentation

A 47-year-old Brazilian female living in Portugal, with no significant medical history, developed proximal muscle weakness in her upper and lower limbs and noticed dark-colored urine one week after recovering from a viral respiratory infection. These symptoms resolved spontaneously within two weeks, and she remained asymptomatic for the next two months. However, she then experienced a recurrence of muscle weakness, accompanied by generalized fatigue. She consulted her family physician, who referred her to internal medicine due to elevated CK and lactate dehydrogenase (LDH) levels. During a trip to Brazil, she was evaluated by a neurologist and prescribed levothyroxine 100 mcg for hypothyroidism, but her symptoms persisted. Following this, she contracted dengue fever, which significantly worsened her muscle weakness, making it difficult for her to maintain an upright position and perform overhead activities with her arms. Upon returning to Portugal, the patient was admitted to the emergency department. Initial examination revealed she was alert and hemodynamically stable, with no skin changes, signs of arthritis, or significant muscle atrophy. Muscle strength was graded IV/V in the lower limbs and III/V in the upper limbs. Laboratory results showed aspartate aminotransferase at 213 U/L, alanine aminotransferase at 368 U/L, and CK at 8,016 U/L. An abdominal ultrasound was unremarkable, and she was subsequently admitted to the Intermediate Medical Care Unit for further monitoring and diagnostic evaluation.

Initial screenings for Epstein-Barr virus, cytomegalovirus, HIV, and hepatitis were negative. Tests for *Treponema pallidum*, *Toxoplasma gondii*, and *Mycobacterium tuberculosis *also returned negative results. Autoimmune screening revealed strongly positive anti-HMGCR antibodies, while antinuclear antibodies, anti-double-stranded DNA, and extractable nuclear antigens were negative. A detailed timeline of laboratory results is provided in Table 1.

**Table 1:** Laboratory results over time ALT, alanine aminotransferase; AST, aspartate aminotransferase; CK, creatine kinase; ESR, erythrocyte sedimentation rate; LDH, lactate dehydrogenase

Test	Day 1 (result)	Day 5 (result)	Day 8 (result)	Day 9 (result)	Day 10 (result)	Day 18 (result)	Day 19 (result)	Normal range
Hemoglobin (g/dL)	14	13.4	12.4	13	-	13.9	12.6	12.5-15.0
Leukocytes (×10^9^)	6,000	6,100	-	12,400	-	-	9,000	4.0-10.0
Lymphocytes (%)	34.10%	42%	-	-	-	-	-	20-40
Platelets (×10^9^)	240	239	249	314	-	322	288	150-400
Iron (µg/mL)	100	-	-	-	-	-	-	33-193
Ferritin (ng/mL)	38.1	-	-	-	-	-	-	30-340
ESR (mm/h)	27	-	80	-	-	-	18	<35
AST (U/L)	168	262	-	615	471	251	128	<40
ALT (U/L)	298	309	-	743	729	598	399	<41
CK (U/L)	5,916	9,717	24,743	14,119	8,416	3,018	1,399	<170
Myoglobin (ng/mL)	2,287	3,792	11,871	6,562	5,545	1,550	715	25-58
LDH (U/L)	954	1,081	1,527	1,317	1,060	548	-	135-245
Troponin T (ng/mL)	296	246	224	149	111	279	245	0-0.01
NT-proBNP (pg/mL)	20	-	94	514	744	-	34	<125
Creatinine (mg/dL)	0.34	0.31	0.23	0.31	0.3	-	0.31	0.5-1.2

**Figure 1:**
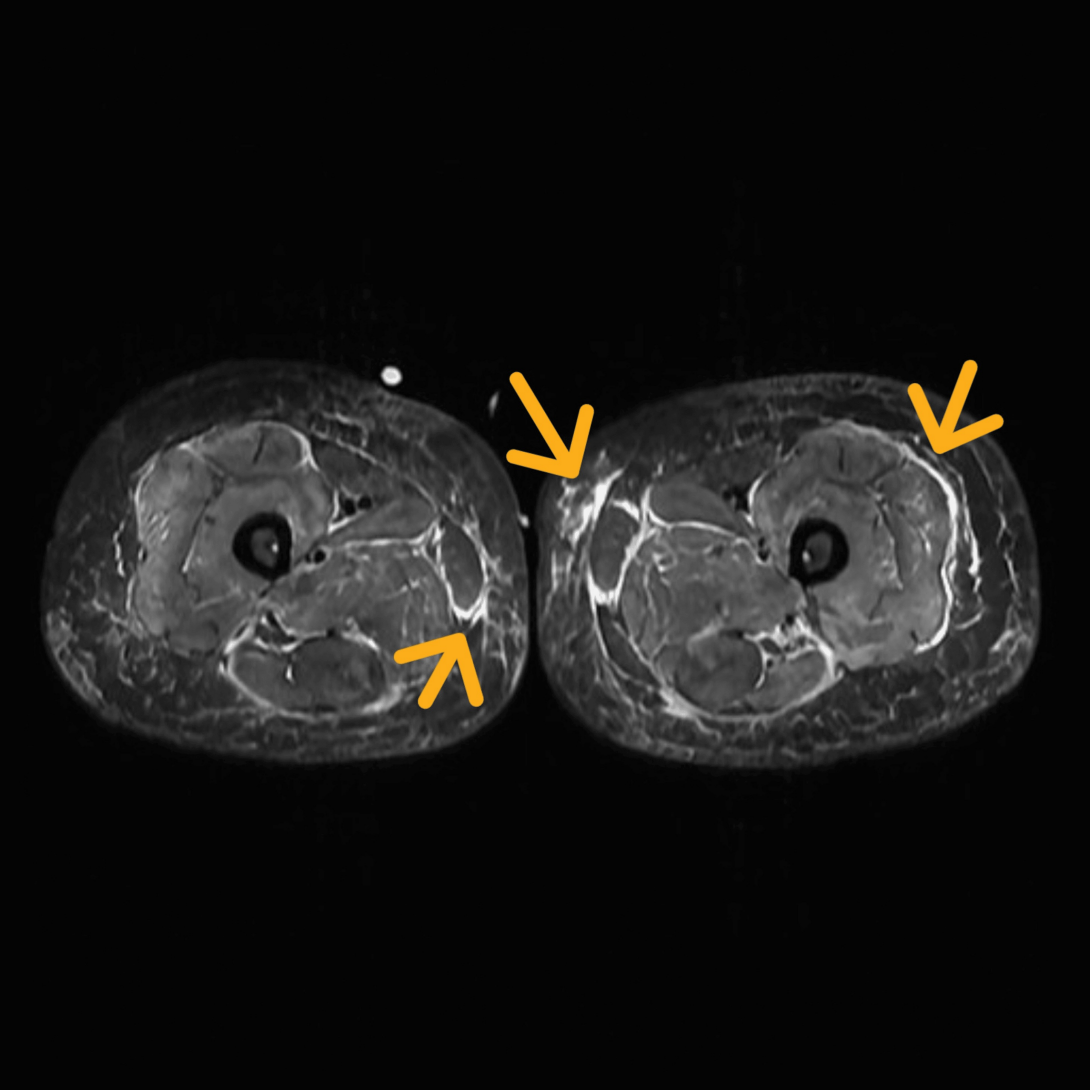
MRI of the muscles of both thighs STIR sequence showing hyperintensity in the thigh and pelvic girdle muscles (yellow arrows). STIR, short tau inversion recovery

**Figure 2:**
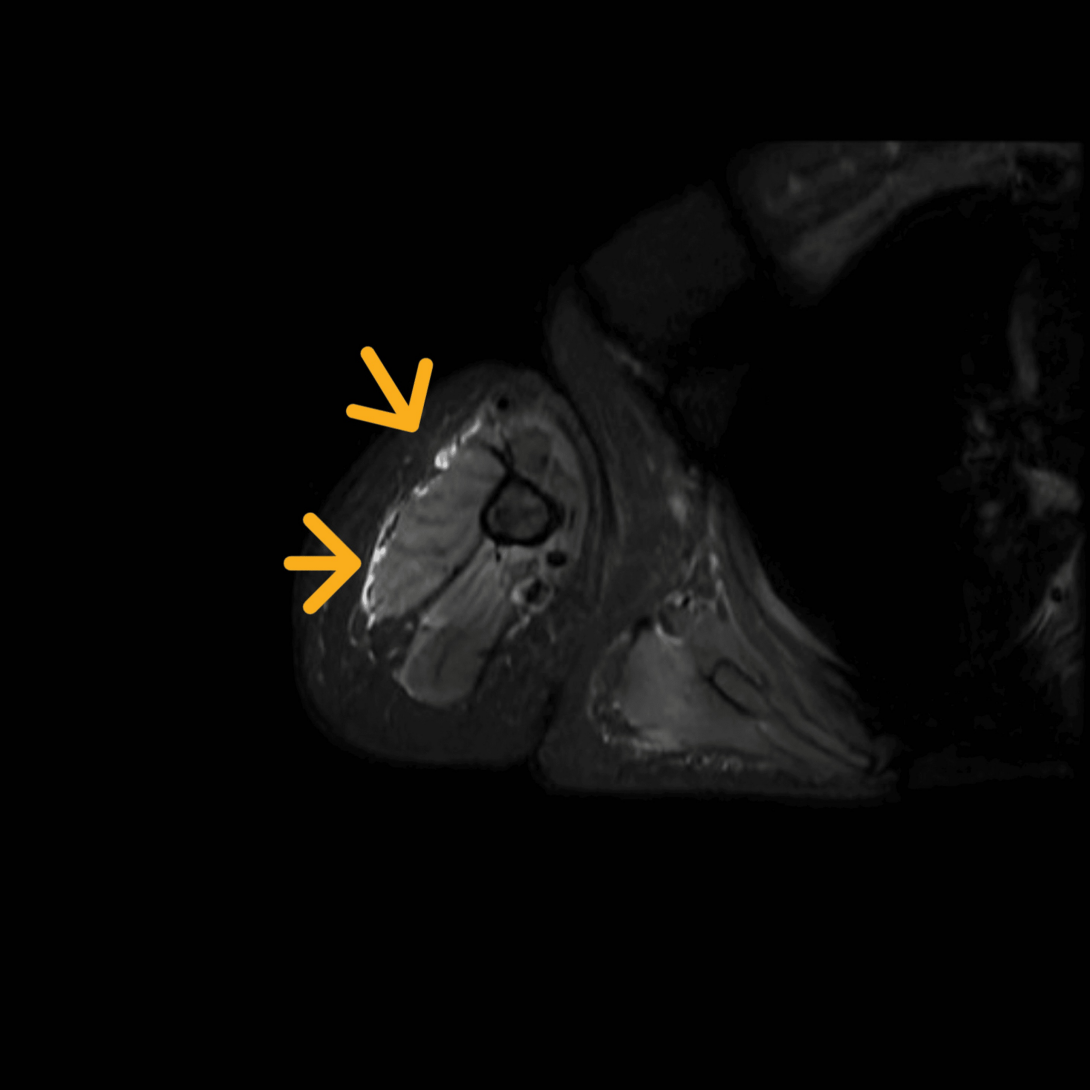
MRI of the muscles of the arm STIR sequence showing hyperintensity in the arm muscles (yellow arrows). STIR, short tau inversion recovery

Given the high suspicion of IMNM, further diagnostic tests were ordered. Electromyography revealed early recruitment and low-amplitude, polyphasic motor units with increased duration, indicative of active inflammatory myopathy. MRI of the thigh and arm showed maintained muscle volumetry, with short tau inversion recovery hyperintensity in the thigh, pelvic girdle, and arm muscles, suggestive of active myositis (Figure 1, Figure 2). A muscle biopsy of the right quadriceps was performed, revealing significant variation in muscle fiber sizes, round shapes of muscle fibers, necrotic fibers, and an absence of inflammatory cell infiltration around the blood vessels on H&E and Gömöri trichrome stains, compatible with necrotizing myopathy. Immunohistochemical staining showed that major histocompatibility complex-1 (MHC-1) was expressed on many myofiber membranes, and CD68-positive cells were predominant.

A muscle biopsy of the right quadriceps was performed, revealing significant variation in muscle fiber sizes, round muscle fiber shapes, necrotic fibers, and an absence of inflammatory cell infiltration around the blood vessels on H&E and Gömöri trichrome stains, which were compatible with necrotizing myopathy. Immunohistochemical staining showed that MHC-1 was expressed on many myofiber membranes, and CD68-positive cells were predominant.

On the fifth day of hospitalization, due to a significant rise in CK levels nearing 10,000 U/L, urine alkalinization with bicarbonate was initiated to prevent acute kidney injury. By the eighth day, the patient’s clinical condition deteriorated, with worsening proximal muscle weakness and an exponential increase in CK to 24,743 U/L, myoglobin to 11,871 U/L, and LDH to 1,527 U/L. Based on the diagnosis of anti-HMGCR-positive necrotizing myopathy, treatment with methylprednisolone at 1 g/day for three days was initiated, followed by intravenous immunoglobulin (IVIG) at 1 g/kg for two days and prednisone at 1 mg/kg/day. Significant clinical and biochemical improvement was observed by the 10th day.

Other investigations included a high-resolution CT scan of the thorax, which was unremarkable. CT scans of the abdomen and pelvis, esophagogastroduodenoscopy, breast ultrasound, mammography, and thyroid ultrasound were all normal, ruling out malignancy. The transthoracic echocardiogram was normal, but cardiac MRI showed diffuse myocardial inflammation suggested by a slight increase in T2 mapping. Due to cardiac involvement, rituximab therapy was initiated on the 18th day. The patient was discharged the following day with instructions for follow-up in the outpatient clinic.

## Discussion

This case of IMNM in a 47-year-old patient, who had no prior statin exposure but presented with a history of viral infection, highlights the complexities involved in diagnosing and treating this rare condition. Notably, the anti-HMGCR antibody was identified in 2010 as a specific marker for a subset of IMNM, particularly in patients with a history of statin use. However, subsequent research has demonstrated its relevance in cases without such exposure, as illustrated in this report [1-3,7-10].

Similar to other autoimmune diseases, a slight female predominance has been observed [8]. Most patients with anti-HMGCR myopathy are adults, with a mean age of 55 years at diagnosis. Conversely, those with non-statin-exposed anti-HMGCR tend to be younger, with a mean age of 40 years at onset [1,8].

Diagnosing IMNM is particularly challenging in patients lacking traditional risk factors such as statin use. The patient initially presented with symptoms that could easily be attributed to other inflammatory myopathies or even infectious myopathies, given her recent viral infections. The association between viral infections and the onset of autoimmune myopathies, including IMNM, is recognized, although the exact pathophysiology remains incompletely understood [1-3,5,6,8-10]. Evidence suggests that viral infections can act as triggers in genetically predisposed individuals through mechanisms such as molecular mimicry, as well as inducing muscle fiber atrophy, inflammation, and necrosis via the complement pathway, macrophage activation, and T cell activation [1,3,9,10]. In this case, the patient experienced significant muscle weakness following a respiratory viral infection, with a marked exacerbation of symptoms after contracting dengue. This indicates that viral infections, such as dengue, may trigger or worsen the autoimmune response associated with IMNM.

Approximately two-thirds of IMNM patients exhibit acute or subacute onset. While proximal limb weakness and elevated CK levels are the hallmark clinical manifestations of anti-HMGCR myopathy, further research is necessary to elucidate the nonskeletal muscle manifestations [1,3,5,6]. Although cardiac involvement is rare, it serves as an important prognostic marker [5,6]. Common findings include elevated troponin T levels, dysrhythmias, and conduction abnormalities [6].

Early diagnosis and intervention are crucial, as delays in treatment can result in progressive muscle weakness and potentially life-threatening complications, including respiratory or cardiac failure [4,6]. Immunomodulation remains the cornerstone of IMNM treatment, with corticosteroids combined with steroid-sparing agents being the first-line approach. Recent recommendations advocate for the upfront use of IVIG in conjunction with corticosteroids for anti-HMGCR-positive IMNM [1,2,4,6]. In this case, the patient was initially treated with methylprednisolone and IVIG, followed by prednisone, leading to significant clinical and biochemical improvement. Although rituximab is more commonly employed in anti-SRP myopathy, it may also be applicable to severe cases of anti-HMGCR, despite a lack of robust data in the literature [2]. Given the patient’s rapid deterioration and strong evidence of cardiac involvement, a more aggressive strategy was implemented, incorporating a third immunomodulatory agent.

## Conclusions

IMNM presents a diagnostic and therapeutic challenge, particularly in patients without traditional risk factors such as statin exposure. This case highlights the importance of recognizing viral infections as potential triggers for IMNM, even in the absence of prior statin use. The identification of anti-HMGCR antibodies was crucial in confirming the diagnosis, emphasizing the need for serological testing in patients suspected of having autoimmune myopathy. Early and aggressive treatment is essential for managing IMNM effectively and preventing severe complications.

In this case, a combination of methylprednisolone, IVIG, and rituximab led to significant clinical improvement, demonstrating the potential benefits of a multimodal immunosuppressive approach, particularly in severe cases. Future research should focus on better understanding the mechanisms linking viral infections to IMNM and optimizing treatment protocols to improve patient outcomes. This case underscores the necessity of a multidisciplinary approach to manage IMNM, ensuring timely diagnosis and intervention to mitigate the disease’s potentially debilitating effects.
